# Clinical Characteristics and Outcomes of Pediatric Traumatic Brain Injury Patients in a Tertiary Regional Trauma Center in the Philippines

**DOI:** 10.1089/neur.2025.0002

**Published:** 2025-03-06

**Authors:** Maurice V. Bayhon, Ibet Marie Y. Sih

**Affiliations:** ^1^ Section of Neurosurgery, St. Luke’s Medical Center and Dr. Paulino J. Garcia Memorial and Medical Research Center, Quezon City, Philippines.; ^2^ Department of Clinical Neurosciences, University of Calgary, Alberta, Canada.; ^3^ Chair and Program Director- Section of Neurosurgery, St. Luke’s Medical Center, Manila, Philippines.; ^4^ Division of Neurosurgery, University of the Philippines- Philippine General Hospital, Manila, Philippines.

**Keywords:** pediatric, Philippines, trauma, traumatic brain injury

## Abstract

**Abstract:**

Traumatic brain injury (TBI) is a leading cause of disability and death in children. Limited data exists on pediatric TBI in Southeast Asia, especially in low- and –middle-income countries. This study investigates the clinical characteristics and outcomes of pediatric TBI patients in a tertiary trauma center in the Philippines. This retrospective cohort study examined pediatric patients (18 years and under) with TBI admitted to a Philippine trauma center from 2021 to 2023. A total of 218 patients were included. Data on demographics, injury mechanisms, TBI severity, cranial imaging, surgical procedures, complications, and discharge outcomes were analyzed.

**Abstract:**

Among the 218 patients, 75% were male, and most were aged 13–18. The primary mechanism of injury was motor vehicle crashes (MVCs), especially in older children (80%). For patients under 5, falls and MVCs were equally common. Most cases were mild (69%), and 40.8% had negative cranial imaging. Epidural hematoma (20%) was the most common abnormal finding. Of the patients, 8% required surgery, with craniotomy for epidural hematoma being the most frequent procedure. Half of those who underwent surgery had neurological deficits, and there was one recorded death. Overall, 86.7% of patients were discharged without complications, though only 34.6% of those with severe TBI had good outcomes. The overall complication rate was 8.7%, with mild neurological deficits being most common. The case fatality rate was 4.6%, with severe TBI showing a higher rate of 30.8%. The majority of patients were male teenagers involved in MVCs. Although most TBIs were mild, there was a notable incidence of severe TBI, particularly with epidural hematoma. These findings suggest higher-impact trauma in the Philippines. The complication and mortality rates align with other studies. Efforts should focus on road safety, traffic law enforcement, and public education. A multi-center prospective study is needed to better understand the factors influencing outcomes in pediatric TBI.

## Introduction

Traumatic brain injury (TBI) is one of the leading causes of mortality and morbidity in the world.^1^ Among the pediatric population, TBI is the most common neurological and neurosurgical cause of death and disability.^2–4^ In a comprehensive global review^5^ that included a few pediatric Asian populations, pediatric TBI represented more than half of pediatric injuries in Iran, around 20% of trauma-related emergency admissions in India, and nearly 30% of pediatric injuries in Korea. The incidence of TBI is very likely underreported as there is a lack of organized data across the world, particularly in low- to middle-income countries (LMICs),^2^ with an even greater gap in Southeast Asia. This study aims to provide data and information collected over a 3-year period on pediatric TBI in a rural setting in the Philippines, an archipelagic country in Southeast Asia.

Demographics such as age and sex are very important in reporting pediatric TBI because the mechanisms of injury vary among different age ranges, and the physiology also differs from infancy to adolescence. Sex also plays a role in response to injury, intervention, and recovery.^2^ Previous studies have shown that males are more susceptible to TBI than females across all pediatric age groups.^3^ A bimodal age distribution is frequently observed, with TBI being more common among very young children (0–2 years old) and older adolescents (15–18 years old).^5^

Several scales have been used to define the severity of TBI, with the Glasgow Coma Scale (CGS) being the most widely accepted classification. Comparative analyses have shown that this scale predicts mortality more accurately than modified models.^3,4^ The classification of TBI is as follows: mild TBI patients have a GCS score of 14–15, moderate TBI patients have a GCS score of 9–13, and severe TBI patients have a GCS score of 3–8. Pediatric patients who are classified as having severe TBI have a high mortality and neurological morbidity risk.^3^ A global review has shown that pediatric TBI patients are mostly mild in severity, with up to 90% of all patients having negative imaging findings.^5^ Only a fraction of patients (<10%) require surgical intervention.^5^

Having a population-specific study is of importance as the primary mechanisms of injury vary worldwide and are heavily influenced by the socio-economic conditions of each region.^2^ Most published work emanates from high-income nations, but the vast majority of cases occur in LMICs.^2,6^ This study is one of the first to present data on pediatric TBI in the Philippines. Understanding the demographics, mechanisms of injury, and outcomes is essential in raising awareness and identifying possible gaps in medical and surgical care for these patients. In addition, gaining insight into the typical clinical course in a rural tertiary hospital set in a developing country can also aid in educating families about prognosis and what to expect during their hospital stay.

## Methods

### Study design

This is a descriptive retrospective cohort study. Data were collected by means of medical record review of patients 18 years old and younger who were admitted due to TBI at a tertiary regional trauma center in the Philippines. The time frame for data collection was from January 2021 to December 2023.

### Participants

All patients with a discharge diagnosis of TBI from the timeframe previously stated were identified. The following inclusion criteria were used: less than or equal to 18 years of age, admitted to the hospital with TBI as a clinical discharge diagnosis with supportive cranial radiographical findings. We excluded patients who had incomplete records and patients who were seen in the emergency room but not admitted. 259 patients were initially screened, out of which 218 patients qualified for review.

### Variables

The following variables were collected: age, sex, mechanism of injury, GCS score, cranial radiographical findings, and surgical procedures done, if any. Clinical outcomes upon discharge, mortality, and complications were also recorded.

### Data sources and measurements

The discharge diagnosis of TBI, along with the corresponding ICD-10 code (International Classification of Diseases),^4^ from the medical records was required for the patient to be included in the review. Data were collected and encoded to ensure confidentiality of patient data. Cranial imaging findings from computed tomography (CT) scans or magnetic resonance imagings (MRIs) were tallied. If a patient had more than one finding on imaging, the most predominant or clinically significant finding was recorded for the purpose of the study. At least two radiologists reviewed the CT scan or MRI findings before documenting it as an official report.

Classification of TBI severity was based on the GCS. Mild TBI patients have a GCS score of 14–15, moderate TBI patients have a GCS score of 9–13, and severe TBI patients have a GCS score of 3–8.

### Bias

The study represented only those who were admitted to the hospital and excluded those who were only seen in the emergency room and sent home. Selection bias may occur because the sample population may not represent the target population of our study. However, enumerating all patients with pediatric TBI in the review lessens the impact of the bias.

### Study size

Sample size was calculated based on the estimation of the population proportion of TBI as a cause of emergency admissions from pediatric injury assumed to be 30%. We used a margin of error of 5% with a confidence level of 90%. The sample size calculated was 229. The number of patients eligible for our study was 218.

### Statistical methods

We used descriptive statistics for data presentation and analysis. Frequency and percentage were used for categorical variables. Mean and standard deviation were used to analyze the continuous variables. The program Statistical Package for the Social Sciences (SPSS) version 27.0 was used to analyze data.

### Ethical considerations

All data were collected by database, records, and chart review. Notes of doctors, nurses, and medical staff were included in data gathering. Only the investigators were allowed to gather patient data inspect and analyze records. The patient’s name remained confidential, and only the data enumerated above were collected and analyzed. Prior to undergoing the treatment, informed consent was obtained from each patient. The study abided by the Principles of the Declaration of Helsinki (2013) and was conducted along the Guidelines of the International Conference on Harmonization-Good Clinical Practice. The clinical protocol and all relevant documents were reviewed and approved by the Institutional Ethics Review Committee. The main person responsible for storage of data was the principal investigator. Data were stored electronically in a password-protected file in the investigator’s personal laptop. Access to the data was limited to the study investigators only.

## Results

### Participants

The majority of the patients were males (75%) between the ages of 13–18 (52.8%), with a mean age of 11.5 ± 5.7 years. Motor vehicle crashes were the most common mechanism of injury in almost 80% of patients, and the majority (69%) of patients sustained a mild TBI (Table 1).

**Table 1.** Demographics, Age Group, Mechanism of Injury, and Presenting Severity of Pediatric Traumatic Brain Injury Patients

Demographic	Total *N* = 218
Sex	
Male	164 (75%)
Female	54 (25%)
Age in years	
<5	32 (14.7%)
5–12	71 (32.6%)
13–18	115 (52.8%)
Mechanism of Injury	
MVC	174 (79.8%)
Fall	43 (19.7%)
Shaken Baby Syndrome	1 (.01%)
Severity	
Mild	151 (69%)
Moderate	41 (19%)
Severe	26 (12%)

*MVC, motor vehicle crash*.

**FIG. 1.**
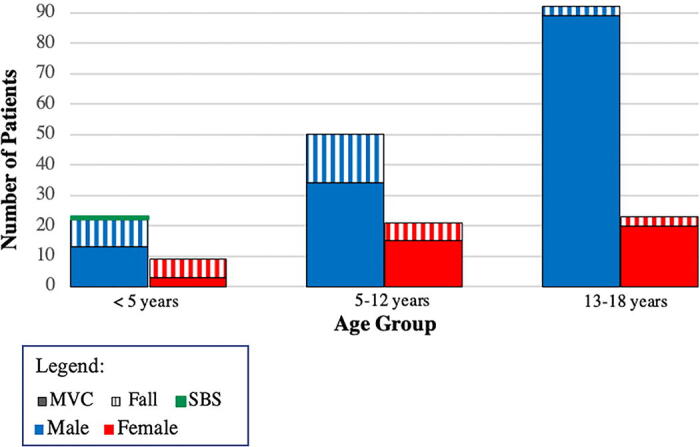
Pediatric traumatic brain injury patients categorized by age group, sex, and mechanism of trauma.

Figure 1 shows that across all age groups, males were consistently more commonly affected than females. Among teenagers aged 13–18, MVCs were significantly more common than falls (96.7% of male patients, 87% of female patients). However, in children under 5 years, there was no significant difference in the incidence of TBIs from MVCs and falls, with falls being more common among females (66.7%). There was only one reported case of non-accidental trauma, specifically Shaken Baby Syndrome, in a 5-month-old male.

### Radiographical findings

The results of the cranial CT scan and MRI procedures performed on admitted pediatric TBI patients were reviewed (Table 2). If a patient had multiple findings on imaging, the most predominant or clinically significant finding was recorded. In our study, 87 patients (40.8%) had negative imaging; the second most common finding was an epidural hematoma at 20% (Table 2).

**Table 2.** Radiographical Findings of Pediatric Patients with TBI

Radiographical finding	*N* = 218 (%)
Negative Imaging (concussion)	89 (40.8)
Epidural Hematoma	43 (20.0)
Subdural Hematoma	22 (10.0)
Contusion Hematoma	20 (9.1)
Skull Fracture	17 (7.8)
Diffuse Cerebral Edema	15 (6.9)
Subarachnoid Hemorrhage	10 (4.6)
Others (IVH, DAI findings, etc.)	2 (0.9)

TBI, traumatic brain injury.

### Surgical outcomes

Table 3 shows that out of 218 patients, 18 (8%) patients underwent surgical intervention. Eleven patients sustained a mild TBI, while four and three patients sustained moderate and severe TBIs, respectively. The most common procedure was craniotomy for epidural hematoma (11 patients, 61.1%), followed by decompressive craniectomy (5 patients, 27.8%). Among the surgical cases, about half were discharged with mild residual neurological deficits (aphasia, confusion, hemiparesis), while 38.9% were discharged with return to baseline function. There was only one mortality recorded among the surgical cases (5.6%).

**Table 3.** Case Severity, Procedure, and Outcomes of Patients with Pediatric Traumatic Brain Injury Who Underwent Surgery

Surgical cases	Total *N* = 18
Severity	
Mild	11 (61.1%)
Moderate	4 (22.2%)
Severe	3 (16.7%)
Procedure	
Craniotomy for Epidural Hematoma	11 (61.1%)
Decompressive Craniectomy	5 (27.8%)
Burr-hole	1 (5.6%)
Ventriculoperitoneal Shunt	1 (5.6%)
Outcome	
Discharged well	7 (38.9%)
Neurological Deficits	10 (50.6%)
Mortality	1 (5.6%)

### Clinical outcomes

Among all patients with pediatric TBI, the majority of patients (86.7%) were discharged well with a return to baseline neurological status, regardless of TBI severity. This positive outcome was evident for patients who sustained mild and moderate TBIs with recovery rates of 97.4% and 80.5%, respectively. The opposite is true for patients who sustained severe TBIs, wherein only 34.6% were discharged in good condition (Table 4).

**Table 4.** Clinical Outcomes of Patients with Pediatric Traumatic Brain Injury

Outcome (%)	TBI severity			
	Mild	Moderate	Severe	Total
	*N* = 151	*N* = 41	*N* = 26	*N* = 218
Discharged well	97.4	80.5	34.6	86.7
All complications	2.0	17.1	34.6	8.7
Neurological deficits	—	17.1	23.1	6.0
Seizure	2.0	—	—	1.4
HIE	—	—	7.7	0.9
Pseudoaneurysm	—	—	3.8	0.5
Mortality	0.7	2.4	30.8	4.6

TBI, traumatic brain injury; HIE, hypoxic ischemic encephalopathy.

The most common complication observed in our study was the presence of neurological deficits such as aphasia, confusion, and hemiparesis (6.0%), followed by post-traumatic seizures and hypoxic-ischemic encephalopathy. One case of traumatic pseudoaneurysm was recorded. Among all patients with pediatric TBI, the overall complication rate was 8.7%. Most of the complications were observed among those who sustained severe and moderate pediatric TBIs at rates of 34.6% and 17.1%, respectively (Table 4).

The overall case fatality rate recorded in our study was 4.6%, with 10 of 218 patients succumbing to their injuries. 8 of 10 deaths were patients with severe TBI who died due to brain herniation and/or diffuse cerebral edema, while the remaining two deaths were patients with mild and moderate TBIs who died due to respiratory failure and pneumonia, respectively. It is important to note the significant case fatality rate among those with severe TBI, which was 30.8%.

## Discussion

This study aims to provide information about the characteristics and clinical outcomes of pediatric TBI patients admitted at a tertiary regional trauma care center in the Philippines, an LMIC in the Southeast Asian region with insufficiently reported data. Most of our pediatric patients were male teenagers (13–18 years) who were involved in a motor vehicular crash. Patients were mostly classified as mild TBI on admission, and the majority were medically managed and discharged with return to baseline neurological function. The most common result on cranial imaging was a negative finding, but among those with pathological radiological findings, acute epidural hematoma was the most common predominant finding.

### Demographics: Age, sex, and mechanism of injury

The results of our study show that the majority of the patients admitted for TBI in pediatrics were males (75%) between the ages of 13 and 18 years old with a mean age of 11.5 ± 5.7 years. Our findings are in contrast to the study of Dewan et. al.,^5^ which reported that more than half of patients were 4 years old or younger in East Asian countries, and a bimodal age distribution among very young children (0–2 years) and adolescents (15–18) was observed globally. The results of our study also show that the most common mechanisms of TBI in pediatrics were motor vehicular crash (80%) and falls (20%), similar to those of previous studies.^5–7^

Teenage males tend to be more involved in high-risk behaviors such as alcoholism, drug abuse, and motorcycle riding. These high-risk behaviors, coupled with the lack of sidewalks, low helmet compliance among motorcyclists, poor highway construction, and low educational attainment leading to poor compliance with road safety regulations, all contribute to the results observed in our study.^8^

We observed that in children under 5 years, TBI due to falls were almost as common as TBI due to MVCs. This is an expected finding since very young children have immature ambulatory skills, disproportionately large heads, and immature neck muscles.^3^ This highlights the importance of further educating and reminding parents and primary caregivers to supervise their children more closely at this age.

### Neurological status

The majority of patients admitted were cases of mild TBI, similar to the findings of a variety of studies. On the other hand, our study identified a higher incidence of severe TBI at 12% in contrast to the 3–7% reported in other reviews.^5^

The most common result on cranial imaging was a negative finding, which was clinically diagnosed as a concussion and often associated with mild TBI. The most common pathological radiological findings identified were epidural hematomas (20%). This finding, combined with the higher incidence of severe TBI in our study, suggests that injuries in our setting were more high-impact and resulted in more devastating consequences.

### Outcomes

For pediatric trauma patients, the presenting GCS score is predictive for mortality and injury outcomes.^9^ In our study, majority of patients admitted with mild or moderate TBI were discharged without neurological deficits. However, our study found a 34.6% complication rate and 30.8% case fatality rate among those with severe TBI. This is similar to modern mortality rates in severe TBI that have been reported to range from 20–39%.^3^

The overall complication and/or disability rate in our study was 8.7%, which is comparable to studies done in Taiwan^10^ and America^11^ with reported rates of 8% and 11%, respectively. Our overall case fatality rate was 4.6%, which is within the mortality rates ranging from 1–7% reported in previous retrospective review studies.^5^

Surgical intervention was warranted in 8% of our patients, which is similar to other studies conducted in Asian lower-middle and upper-middle-income countries^12–14^ with a surgical intervention rate ranging from 6% to 14%. Among all patients who underwent surgery in our study, about 50% were discharged with mild to moderate neurological deficits, and 5.6% died. These findings are comparable to those of a Taiwanese study^10^ that reported a lower rate of mild to moderate neurological deficits at 28%, but a higher case fatality rate at 8% among pediatric TBI patients who underwent surgery.

### Limitations of the study

The results of the study were based on a single tertiary center. We were dependent on the data that were documented by doctors, nurses, and medical staff. Our study is mainly descriptive and retrospective in nature. Due to poor follow-up compliance, only clinical outcomes and complications that occurred during the duration of hospital admissions were recorded. A multi-center study with a larger population and long-term follow-up is needed to develop a regression model and identify the factors that contribute to improved outcomes of pediatric TBI in Filipino patients.

## Conclusion

Most of our pediatric TBI patients were male teenagers (13–18 years) involved in MVCs. This is in contrast to previous Asian studies that have reported most patients being in early childhood years (0–4 years). The majority of our patients sustained a mild TBI. We observed a higher incidence of severe TBI compared with other studies, and among patients with pathological cranial imaging, epidural hematoma was the most common predominant finding, suggesting that accidents in the Philippine setting tend to be more high-impact, resulting in more severe injuries.

With regards to clinical outcomes, majority of pediatric patients with mild or moderate TBI were discharged in good condition without neurological deficits. Our complication rates, surgical intervention rates, and mortality rates were consistent with those reported in other studies. It appears that future efforts to reduce the morbidity and mortality from pediatric TBI among Filipino patients should primarily focus on improving road safety conditions, enforcing traffic laws, and enhancing traffic safety education.

## Abbreviations Used

CT: Computed Tomography
DAI: Diffuse Axonal Injury
GCS: Glasgow Coma Scale
ICD: International Classification of Disease
IVH: Intraventricular Hemorrhage
LMICs: Low to Middle Income Countries
MRI: Magnetic Resonance Imaging
MVC: Motor Vehicular Crash
SPSS: Statistical Package for the Social Sciences
TBI: Traumatic Brain Injury

## References

[1] Najem D, Rennie K, Ribecco-Lutkiewicz M, et al. Traumatic brain injury: Classification, models, and markers. Biochem Cell Biol 2018;96(4):391–406; doi: 10.1139/bcb-2016-0160 29370536

[2] Figaji A. An update on pediatric traumatic brain injury. Childs Nerv Syst 2023;39(11):3071–3081; doi: 10.1007/s00381-023-06173-y 37801113 PMC10643295

[3] Haydel MJ, Weisbrod LJ, Saeed W., (eds). Pediatric Head Trauma. In StatPearls. StatPearls Publishing; 2024. 30725714

[4] Mena JH, Sanchez AI, Rubiano AM, et al. Effect of the Modified Glasgow Coma Scale Score Criteria for Mild Traumatic Brain Injury on Mortality Prediction: Comparing Classic and Modified Glasgow Coma Scale Score Model Scores of 13. J Trauma 2011;71(5):1185–1192; discussion 1193; doi: 10.1097/TA.0b013e31823321f8 22071923 PMC3217203

[5] Dewan MC, Mummareddy N, Wellons JC, et al. Epidemiology of Global Pediatric Traumatic Brain Injury: Qualitative Review. World Neurosurg 2016;91:497–509.e1; doi: 10.1016/j.wneu.2016.03.045 27018009

[6] Dewan MC, Rattani A, Gupta S, et al. Estimating the global incidence of traumatic brain injury. J Neurosurg 2019;130(4):1080–1097; doi: 10.3171/2017.10.JNS17352 29701556

[7] Balogun JA, Koko AM, Adebayo A, et al. Fall-related traumatic brain injury in a Nigerian pediatric population. J Clin Neurosci 2023;109:26–31; doi: 10.1016/j.jocn.2023.01.007 36642033

[8] Nantulya VM, Reich MR. The neglected epidemic: Road traffic injuries in developing countries. Bmj 2002;324(7346):1139–1141; doi: 10.1136/bmj.324.7346.1139 12003888 PMC1123095

[9] Cicero MX, Cross KP. Predictive value of initial Glasgow coma scale score in pediatric trauma patients. Pediatr Emerg Care 2013;29(1):43–48; doi: 10.1097/PEC.0b013e31827b52bf 23283262

[10] Tsai W-C, Chiu W-T, Chiou H-Y, et al. Pediatric traumatic brain injuries in Taiwan: An 8-year study. J Clin Neurosci 2004;11(2):126–129; doi: 10.1016/S0967-5868(03)00156-5 14732368

[11] Reid SR, Roesler JS, Gaichas AM, et al. The epidemiology of pediatric traumatic brain injury in Minnesota. Arch Pediatr Adolesc Med 2001;155(7):784–789. 11434844 10.1001/archpedi.155.7.784

[12] Chan HC, Aasim WAW, Abdullah NM, et al. Characteristics and clinical predictors of minor head injury in children presenting to two Malaysian accident and emergency departments. Singapore Med J 2005;46(5):219–223. 15858690

[13] Işık HS, Gökyar A, Yıldız O, et al. Pediatric head injuries, retrospective analysis of 851 patients: An epidemiological study. Ulus Travma Acil Cerrahi Derg 2011;17(2):166–172; doi: 10.5505/tjtes.2011.22800 21644096

[14] Agrawal A, Agrawal CS, Kumar A, et al. Epidemiology and management of pediatric head injury in eastern Nepal. Afr J Paediatr Surg 2008;5(1):15–18; doi: 10.4103/0189-6725.41630 19858657

